# Alternatively Constructed Estrogen Receptor Alpha-Driven Super-Enhancers Result in Similar Gene Expression in Breast and Endometrial Cell Lines

**DOI:** 10.3390/ijms21051630

**Published:** 2020-02-27

**Authors:** Dóra Bojcsuk, Gergely Nagy, Bálint László Bálint

**Affiliations:** 1 Genomic Medicine and Bioinformatic Core Facility, Department of Biochemistry and Molecular Biology, Faculty of Medicine, University of Debrecen, 4032 Debrecen, Hungary; bojcsuk.dora@med.unideb.hu; 2 Doctoral School of Molecular Cell and Immune Biology, Faculty of Medicine, University of Debrecen, 4032 Debrecen, Hungary; 3 Department of Biochemistry and Molecular Biology, Faculty of Medicine, University of Debrecen, 4032 Debrecen, Hungary

**Keywords:** estrogen receptor alpha (ERα), transcription factor, enhancer, super-enhancer, MCF-7 cell line, Ishikawa cell line, ChIP-seq

## Abstract

Super-enhancers (SEs) are clusters of highly active enhancers, regulating cell type-specific and disease-related genes, including oncogenes. The individual regulatory regions within SEs might be simultaneously bound by different transcription factors (TFs) and co-regulators, which together establish a chromatin environment conducting to effective transcription. While cells with distinct TF profiles can have different functions, how different cells control overlapping genetic programs remains a question. In this paper, we show that the construction of estrogen receptor alpha-driven SEs is tissue-specific, both collaborating TFs and the active SE components greatly differ between human breast cancer-derived MCF-7 and endometrial cancer-derived Ishikawa cells; nonetheless, SEs common to both cell lines have similar transcriptional outputs. These results delineate that despite the existence of a combinatorial code allowing alternative SE construction, a single master regulator might be able to determine the overall activity of SEs.

## 1. Introduction

Estrogen receptor alpha (ERα) is a well-studied member of the nuclear receptor (NR) superfamily and functions as a master regulator in several cell types, including breast, ovarian, and endometrial cancer cells [1,2,3]. The continuously changing level of its natural ligand, 17β-estradiol (E2), is indispensable for maintaining the ovarian cycle, and although normal hormone levels are responsible for female characteristics and contribute to healthy bone density among others, a higher estrogen/progesterone ratio in breast and endometrial tissues is linked to an increased risk of breast and endometrial cancer in postmenopausal women [4,5,6].

DNA binding by the receptor/ligand complex can occur directly through the estrogen response element (ERE) or via indirect protein-protein interactions [7,8,9,10,11]. According to previous studies, nearly one million EREs occur in the human genome, but only a small fraction of these EREs are located within open chromatin regions [12,13]. In addition, the number of EREs bound by ERα is reduced because the affinity of ERα for different EREs exhibits a broad spectrum [14]. High-affinity elements or canonical elements can immobilize the transcription factor (TF), thus exerting a much greater influence on gene expression than the weaker elements that most likely need other stabilizing TFs to generate the activator complex or indirect DNA-protein interactions where an association with other TFs is required [15,16].

In the vicinity of highly expressed genes, multiple TFs are recruited to activate clusters of enhancers within a relatively small stretch of DNA, which are also known as super-enhancers (SEs) or clustered enhancers [17,18]. SEs can be characterized by a combination of active and open chromatin/enhancer marks, such as DNase I hypersensitivity, recruitment of the histone acetyltransferase P300, histone modifications such as acetylation at the lysine 27 residue of the histone H3 protein (H3K27ac), the chromatin modification reader BRD4 (a member of the bromodomain and extraterminal (BET) domain family) that recognizes acetylated histones, and MED1, which is one of the most important subunits of the Mediator complex that has a bridging role between enhancers and promoters via chromatin looping [19,20,21]. Recent studies reinforced that BRD4 and MED1 condensate at SEs in cell nuclei, and SE condensates can be isolated by liquid-liquid phase separation [22,23]. Interestingly, these protein clusters are assembled in a non-canonical way through the intrinsically disordered domains of their constituents, highlighting the importance of non-classical domain-based protein interactions, although according to several studies, SEs are located within CTCF-demarcated chromatin domains [24,25,26,27]. This phenomenon indicates that SEs concentrate the most important co-activator proteins, which distinguish them from typical enhancers. Although SEs can make physical contact with their target promoters from very long distances, these are mostly identified near cell type-specific or disease-related genes, which act as determinants of cell identity [17,24]. As suggested by several studies on cancer cells, SEs contribute to the expression of relevant biomarker genes that might be used for targeted therapeutic development; however, numerous questions regarding their exact gene regulatory functions remain unanswered [28,29].

We previously presented that the formation of ligand-inducible ERα-driven SEs depends on the presence of canonical EREs, which can initiate further ERα binding events at nearby and even less specific binding sites [15,16]. These primary “mother” or “hub” enhancers (a term coined by Huang et al.) represent active chromatin regions even in the absence of specific treatment, and these form the basis of ligand-inducible SEs [30]. In the present study, we investigated whether (i) ERα-driven SEs develop around the same canonical elements or in the same genomic regions in different cancer cell lines, and also studied (ii) the common and (iii) cell line-specific characteristics of these SEs. Furthermore, we examined in detail whether (iv) the effect of SEs on gene expression is equivalent in two inherently different cancer cell lines. In order to answer these questions, by using publicly available chromatin immunoprecipitation followed by sequencing (ChIP-seq) data, we predicted ERα-driven SEs in human breast cancer-derived MCF-7 and endometrial cancer-derived Ishikawa cell lines in which ERα is a key master regulator. We raised the possibility that comprehensive characterization of cell line-specific ERα-driven gene regulatory units may also be important in understanding how ERα, as a master TF can fine-tune different cell functions in a way that a core set of SEs can retain the same function with different regulatory elements in different tissues.

## 2. Results

### 2.1. General Comparison of the Putative ERα Binding Sites in MCF-7 and Ishikawa Cells

**Figure 1:**
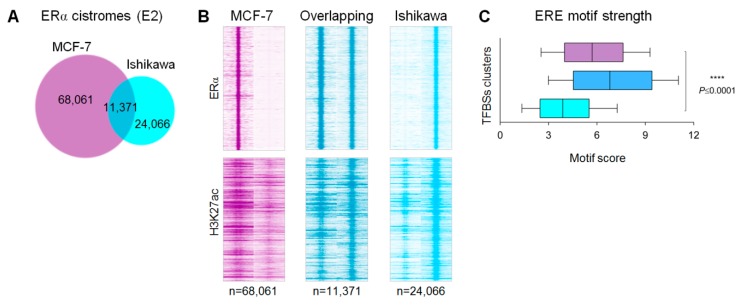
Genome-wide comparison of ERα cistromes and transcriptomes in MCF-7 and Ishikawa cells upon E2 treatment. **(A)** Area-proportional Venn diagram showing the overlap between all ERα TFBSs upon E2 treatment in MCF-7 and Ishikawa cells. **(B)** Read distribution plots showing the presence or absence of the identified cell line-specific and overlapping ERα TFBSs and the H3K27ac signals in 2-kb frames. **(C)** Box plots showing the ERE motif strength within the summit ±100-bp region of the shared and cell line-specific TFBSs, defined in Figure 1A. The boxes represent the first and third quartiles, the lines indicate the median scores and the whiskers indicate the 10th to 90th percentile ranges. Unpaired t-test, **** significant at *p* <0.0001.

Comparing different cell types, enhancers show an extremely high level of variability [31]. By testing the similarities between MCF-7 and Ishikawa cells at the level of ERα cistromes upon E2 treatment, we found that both cell lines had tens of thousands of ERα transcription factor binding sites (TFBSs), but most of these sites were characteristic of only one of the investigated cell lines (Figure 1A). This cell line-specific and overlapping ERα binding pattern is also followed by the presence of H3K27ac signals suggesting that there is a large difference between the chromatin accessibility in the two investigated cell lines (Figure 1B). In line with this result, EREs, which were commonly occupied by ERα in both cell lines, showed the highest motif scores, followed by those of MCF-7-specific and then Ishikawa-specific sites (Figure 1C). Based on our previous findings, these results suggest that a subset of EREs among commonly used EREs might be considered common nucleating sites of SEs and in Ishikawa cells, the lower affinity of ERα-occupied EREs requires the assistance of collaborating factors [15,16].

### 2.2. ERα-driven SE Constituents have Different Motif Preferences in MCF-7 and Ishikawa Cells

**Figure 2:**
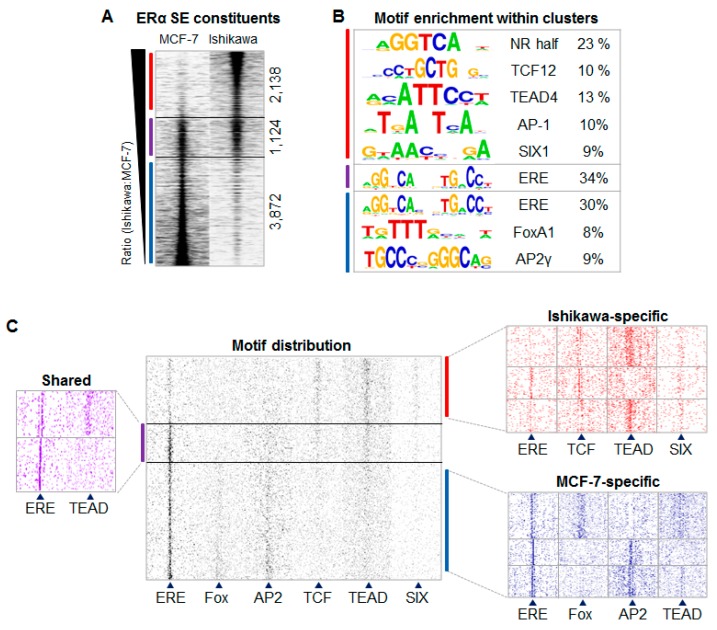

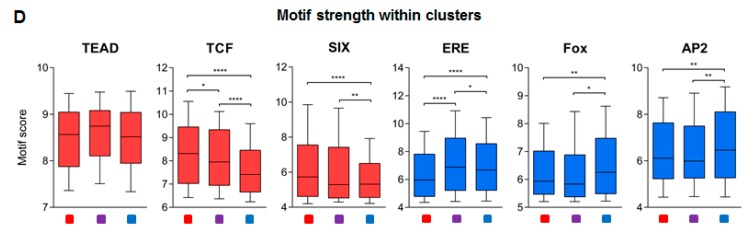
ERα-driven super-enhancer constituents show distinct binding patterns and motif preferences in MCF-7 and Ishikawa cells. (**A**) Read distribution plot showing ERα density on ERα-driven super-enhancer (SE) constituents derived from MCF-7 and Ishikawa cells in 2-kb frames. Peaks were sorted based on the ratio of RPKM (reads per kilobase per million mapped reads) values calculated from Ishikawa and MCF-7 cells and were separated into three different clusters: the red line represents Ishikawa-specific constituents (*n* = 2138), the purple line represents shared constituents (*n* = 1124), and the blue line represents MCF-7-specific SE constituents (*n* = 3872). (**B**) The enriched motifs and their percentages within the target regions of the three clusters. (**C**) The motif distribution plot of ERE, Fox, AP2, TCF, TEAD, and SIX motifs in 1.5-kb frames around the summit position of ERα-driven SE constituents in the same order as introduced in Figure 2A (middle). Colored heat maps represent shared and cell line-specific clusters when peaks were further clustered based on the presence or absence of the most frequent motifs. (**D**) Box plots showing the distribution of motif strengths within the three main clusters introduced in Figure 2A. The boxes represent the first and third quartiles, the horizontal lines indicate the median scores and the whiskers indicate the 10th to 90th percentile ranges. Paired t-test, * significant at *p* < 0.05, ** at *p* < 0.01, *** at *p* < 0.001, **** at *p* < 0.0001.

In accordance with the preliminary findings showing that a subset of enhancers are commonly occupied by ERα in both cell lines, we narrowed our focus on how ERα-driven SEs in different TF environments are assembled; therefore, we assessed ERα binding sites at the SE regions specific for MCF-7 and Ishikawa cells. Importantly, some studies define SEs based on H3K27ac or MED1 signals; here we consider this approach based on the binding density of ERα. We predicted 392 SE regions in MCF-7 and 618 in the Ishikawa cell line respectively, and most of their constituents were characteristic of only one investigated cell line (Figure 2A, Supplementary Figure S1A,B and Table S1A). The cell line-specific, ERα-driven SE constituents were ~3.4 times more abundant in MCF-7 (*n* = 3872) and ~1.9 times more abundant in Ishikawa (*n* = 2138) cells than those present in both cell lines (*n* = 1124) (Figure 2A, Supplementary Figure S1B). The presence of DNase I hypersensitivity, H3K27ac and P300 also followed the three well-separated binding patterns (Supplementary Figure S1C and Table S1B). The resulted “clusters” were referred to as: (1) MCF-7-specific, (2) shared, as they are common to both cell types, and (3) Ishikawa-specific, highlighted if possible in blue, purple, and red, respectively, in the figures.

The first substantial difference observed between the three identified clusters was seen in their enriched DNA motifs (Figure 2B, Supplementary Figure S1D). Within the commonly occupied TFBSs, only the ERE and different direct repeats (DRs) of the nuclear receptor half-site (NR half) were enriched, whereas, in the cell line-specific clusters, motifs of other TFs could also be identified. Specifically, motifs of the Fox and AP2 proteins were enriched in the MCF-7-specific cluster, and motifs of the TEAD, TCF, AP-1, and SIX proteins were enriched in the Ishikawa-specific cluster. The latter cluster did not show enrichment of the ERE motif but only the more general NR half-site, which suggests that in the Ishikawa-specific sites ERα needs the assistance of its co-factor(s).

In MCF-7 cells, in addition to ERα, forkhead box A1 (FoxA1) is the most influential TF and is present in approximately half of ERα-bound genomic regions even in the absence of E2 [32,33]. FoxA1 plays a role as a pioneer factor of ERα, thus facilitating its binding, while activator protein 2 gamma (AP2γ), another major TF, stabilizes the DNA-protein interaction [34]. Although GATA binding protein 3 (GATA3) and retinoic acid receptor alpha (RARα) were also identified as ERα cooperative factors in breast cancer cells, these were not identified as crucial players in our investigations on ERα-driven SEs [15,35,36,37].

All TF families that had their motifs enriched at the sites bound by ERα identified in Ishikawa cells were previously directly associated with ERα in endometrial cancer [38]. TEA domain transcription factor 4 (TEAD4) cooperates with activator protein 1 (AP-1) in the chromatin of endometrial cancer cells and establishes an interaction between ERα and progesterone receptor (PR) in breast cancer cells [39,40], transcription factor 12 (TCF12), also known as HEB, functions as an oncogene as well as a tumor suppressor in several human cancer types [41]. In mouse experiments, TCF12 was also identified as a key prognostic factor for endometrial cancer [42]. Although all of the above-mentioned endometrial cancer-related proteins follow the binding of ERα in Ishikawa cells, there are no reports on these proteins regarding ERα-driven SEs. Furthermore, the elevated expression of SIX1 is a biomarker of hyper- or dysplastic cells in human endometrial cancers [43]. The exclusivity of the motifs in the cell line-specific clusters suggested a different mode of action between the SEs and the assembled TF complexes of our two chosen models where ERα is present as a master regulator.

Based on these initial findings, we carried out a detailed investigation of how different TFs contribute to the formation of both cell line-specific and shared ERα-driven SEs. First, for validation, we mapped the matrix of the identified DNA motifs and found that the shared ERα binding sites showed large numbers of ERE and smaller numbers of other elements, whereas the cell line-specific enhancers showed expected motif distribution patterns: Fox and AP2 motifs were enriched at MCF-7-specific ERα binding sites, while TCF, TEAD, and SIX motifs were enriched at Ishikawa-specific ERα binding sites (Figure 2C, Supplementary Figure S2A). Further dividing the three clusters based on motif distribution showed that certain motifs (e.g., ERE and TEAD or Fox and AP2) mutually exclude each other (Figure 1C). In order to examine the strengths of the enriched motifs, we plotted the motif scores within the cell line-specific and shared clusters (Figure 2D, Supplementary Figure S2B). 

Generally, motif strengths correlated well with motif distribution patterns; however, TEAD motifs did not show significantly different motif strengths between the analyzed clusters (*p* > 0.05; not significant). These results indicated that the two cell lines use different sets of TFs and that motif distribution within SEs may represent a DNA-encoded component of SE-driven transcription regulation.

### 2.3. Response Elements Determine the Hierarchy between FoxM1/TCF12/TEAD4 Proteins in Ishikawa Cells

As TF motifs may be bound by several proteins of a TF family, we compared the expression levels of all members of the emerging TF families from publicly available RNA-seq data sets. In addition to *FOXA1* and *TFAP2C* (encoding AP2γ), *ESR1* (encoding ERα) also showed much lower expression in Ishikawa cells than in MCF-7 cells. This result is consistent with the less prominent dominance of ERα in Ishikawa cells than in MCF-7 cells and partially consistent with substitution or supplementation of its function by other TFs (Figure 1B). Of the more than 40 members of the Fox family, *FOXM1* had the highest expression in Ishikawa cells; however, *FOXD1* also showed a notable expression level (Supplementary Figure S3A,B and Table S1B). Based on previous studies, in Ishikawa cells, forkhead box M1 (FoxM1) and CCAAT/enhancer-binding protein beta (C/EBPβ) were also described as potential regulatory partners of ERα [38]. Although the C/EBP motif—similarly to the Fox motif—is not a typical component of ERα-driven SEs, previous studies suggest that C/EBPβ can stabilize ERα in a DNA binding complex; therefore, we plotted the gene expression levels of the whole gene family (Supplementary Figure S3C) [44]. *TCF12* and *TEAD4* showed higher expression in Ishikawa cells than in MCF-7 cells, but this pattern was also observed for other family members, such as *TCF3* and *TEAD2*. Although *SIX* genes were expressed at low levels in both cell lines, *SIX5* was expressed at higher levels in Ishikawa cells, and *SIX4* was specific to MCF-7 cells. Of all these genes, *CEBPB* showed the highest expression, and the genomic distribution of its protein product as investigated by TF mapping followed the binding pattern of ERα even in the absence of ligand treatment in Ishikawa cells (Supplementary Figure S3D); therefore, we included it in additional analyses. In accordance with the absence of any C/EBP motif enrichment in our previous results (Figure 2B, Supplementary Figure S1E), we could not detect any C/EBP motif enrichment around the ERα SE constituents (Supplementary Figure S3E). At these sites, similar to FoxM1, C/EBPβ may bind indirectly to the DNA through ERα or other DNA binding collaborators less affected by E2 treatment. Together with all of the above-mentioned TFs, C/EBPβ followed cell line-specific ERα binding patterns (Supplementary Figure S3D,F). The gene expression comparison confirmed the role of collaborating TFs and highlighted FoxM1 as a TF with a major role in Ishikawa cells.

**Figure 3:**
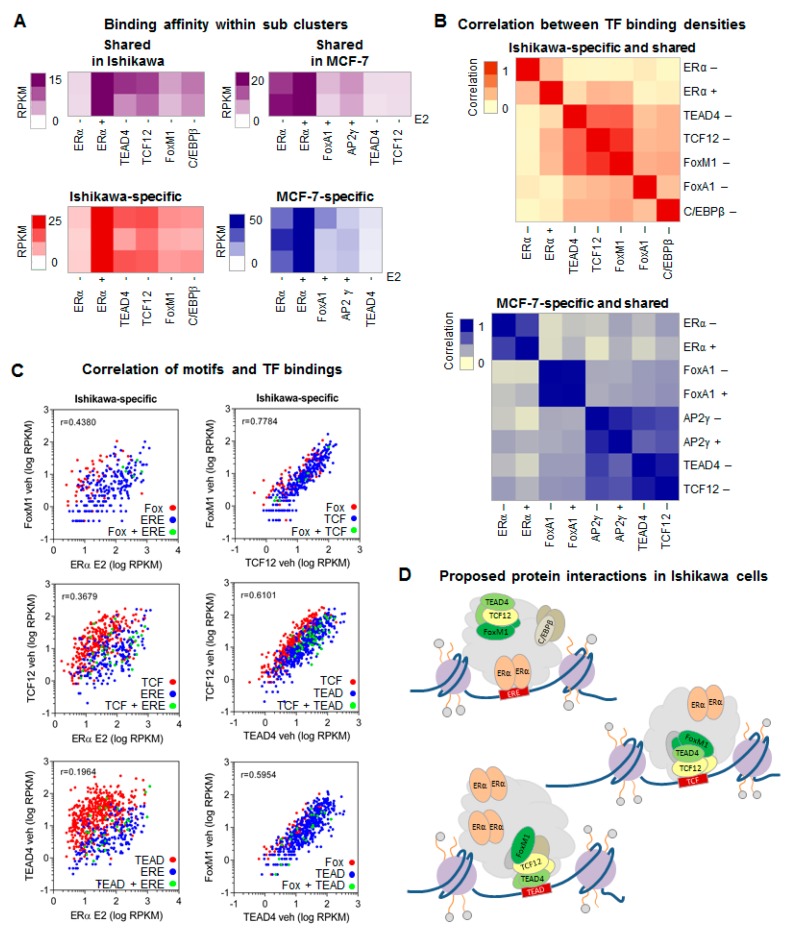
Response elements determine a consistent hierarchy between transcription factor binding events. (**A**) Heat maps depict the density of relevant TFs in the presence (+) or absence (−) of E2 within the same sub-clusters introduced in Figure 2C. The plotted densities are the averages of the values calculated by HOMER within 50-bp regions around the summit of the sub-clusters’ SE constituents. In the case of shared peaks, ChIP-seq coverages were separately calculated for both Ishikawa and MCF-7 cells. (**B**) Correlation plots showing the correlation coefficients (r) calculated from the densities of all investigated TFs on the SE constituents (summit ± 50-bp regions) of Ishikawa and MCF-7 cells. (**C**) Scatter plots showing the densities of the indicated TFs (upon vehicle [veh] or E2 treatment) on their DNA binding motifs within the MCF-7- or Ishikawa-specific ERα-driven SE constituents. Red and blue dots represent protein binding on a specific single motif, and green dots represent protein binding on a region with the motifs of both examined TFs. (**D**) Working models of the supposed hierarchy between ERα, FoxM1, TCF12, and TEAD4 TFs in Ishikawa cells based on the presence of ERE, TCF, or TEAD response elements.

In addition, by examining the overall binding of specific TFs to DNA as expressed in average RPKM values per motif distribution-based sub-clusters defined in Figure 2C, we identified specific patterns of protein co-occurrences. All TFs showed high density at their specific elements and to a lesser extent, other TFs were also recruited, except for FoxA1 and AP2γ, which excluded each other’s presence at MCF-7-specific sites (Figure 3A). Unsurprisingly, the recruitment of ERα upon E2 treatment was observed in each sub-cluster even at binding sites that lacked ERE (Figure 3A, Supplementary Table S1B).

In order to closely examine the protein-protein interactions suggested by these results, we performed a correlation analysis on TF binding (Figure 3B). In Ishikawa cells, FoxM1 and TCF12 showed the strongest co-occurrence with each other and with TEAD4 and E2-induced ERα. Although C/EBPβ could be detected at ERα-driven SE constituents, its density did not show any correlation with ERα binding; instead, its density was correlated with the Ishikawa-related FoxM1/TCF12/TEAD4 protein trio. The correlation heat map for MCF-7 suggested more independent recruitment of key TFs.

In order to examine both protein-protein and DNA-protein concomitance, we plotted TF densities at their putative TFBSs (Figure 3C, Supplementary Figure S4A,B). This type of representation clearly visualized that different TFs show higher density at their own elements, and we also obtained information about their co-occurrence with each other at the same genomic sites. In Ishikawa cells, the presence of ERα correlated best with that of FoxM1, followed by co-occurrence with TCF12 and TEAD4. Pairwise comparisons suggested a FoxM1/TCF12, TCF12/TEAD4, and FoxM1/TEAD4 interaction, which implies a tripartite complex interacting with ERα (Figure 3C,D, Supplementary Figure S4A,B). The TCF12/TEAD4 relationship was also reproduced even with lower protein levels in MCF-7 cells (r = 0.8833) (Supplementary Figure S4B). C/EBPβ binding showed no or only weak correlation (r = 0.3489) with other TFs except for FoxM1; however, the low number of response elements could cause a bias towards their interaction (Supplementary Figure S4A). Contact between a steroid hormone receptor and Fox protein is not unprecedented as androgen receptor (AR) and FoxA1 associate to occupy ARE::Fox composite elements. However, a single ARE or Fox motif is usually sufficient for binding by both proteins [45,46,47]. In our proposed mechanism, any TF can bind its DNA element, and this process is sufficient for anchoring the entire complex of proteins. As Ishikawa-specific Fox motifs are very rare (as shown on Figure 2C), FoxM1 is typically not a direct DNA binder. This finding also indicates no need for direct DNA binding by ERα for regulation in Ishikawa cells (unlike our previously described finding in MCF-7 cells) (Figure 3A,B). In MCF-7 cells, there was no close correlation between dominant proteins, and ERα/FoxA1 concomitances seemed to be the least frequent (r = 0.3626). Upon E2 treatment, we observed a slight recruitment of FoxA1 and stronger recruitment of AP2γ, which was previously described (Supplementary Figure S4B). In a few TFBSs, two motifs could be mapped (green dots); these regions were usually bound by their specific TFs to a similar extent.

Together, these findings indicated that TF binding events followed the DNA motif pattern and that well-defined cooperativity and hierarchy existed between TFs promoting the formation of complexes on SEs.

### 2.4. Overlapping SE Regions Are Composed of Different ERα Binding Sites

**Figure 4:**
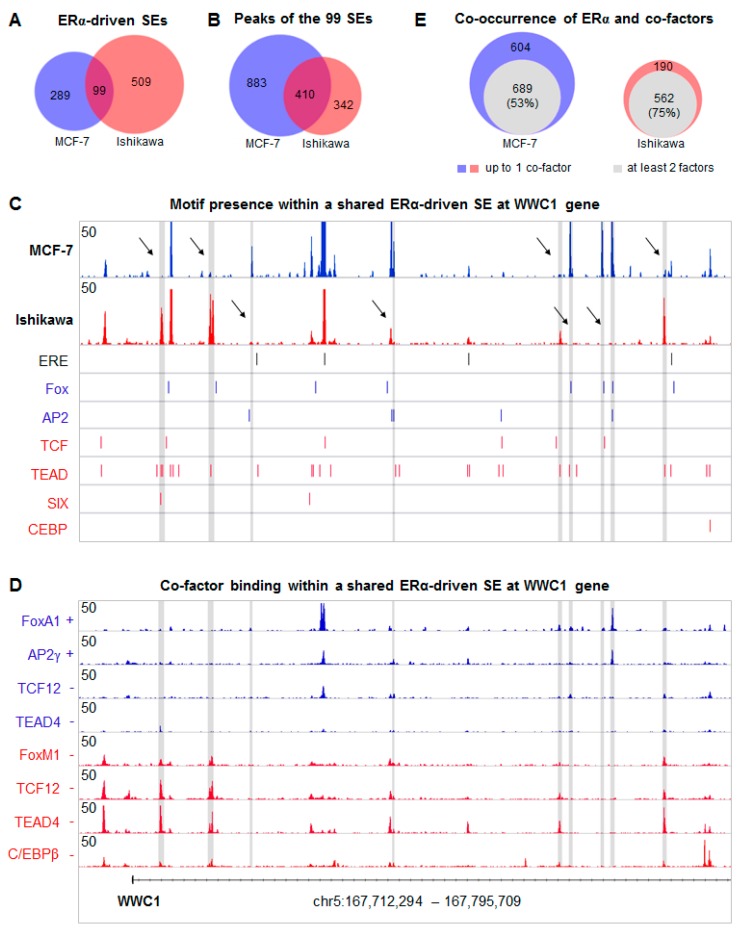
Shared ERα-driven super-enhancers are composed of different transcription factor binding sites in MCF-7 and Ishikawa cells. (**A**,**B**) Area-proportional Venn diagrams representing the overlap between all ERα-driven SEs of MCF-7 and Ishikawa cells (A) and the overlap between the constituents of the 99 shared SEs (**B**). (**C**) The Integrative Genomics Viewer (IGV) snapshot of ERα ChIP-seq coverage on the *WWC1* locus showing a SE that is formed upon E2 treatment in both MCF-7 and Ishikawa cells. The interval scale is 50. The matrix of ERE, Fox, AP2, TCF, TEAD, SIX, and CEBP motifs was mapped within the summit ± 50-bp regions of the ERα peaks, and their putative elements were represented as thin lines (bottom part of the panel). Peaks marked with arrows and highlighted in grey show different binding patterns between MCF-7 and Ishikawa cells. (**D**) The IGV snapshot representing FoxA1, AP2γ, TCF12, and TEAD4 ChIP-seq coverage in MCF-7 and FoxM1, TCF12, TEAD4, and C/EBPβ ChIP-seq coverage in Ishikawa cell line upon vehicle [**−**] or E2 [+] treatment. The interval scale is 50, in all cases. (**E**) Area-proportional Venn diagrams show the proportion of the total 1,293 MCF-7 and 752 Ishikawa-specific ERα peaks (defined on panel B) that show co-occurrence with at least two other TFs.

By focusing on complete ERα-driven SE regions, we found 99 SEs that partly or fully overlapped between MCF-7 and Ishikawa cells. Nonetheless, these “common” SEs shared only a quarter of their ERα TFBSs (410 in total) (Figure 4A,B, Supplementary Figure S5A). This heterogeneity of SE composition, which was also observed at the WWC1 locus, suggests a strong cell type- and TF-specific enhancer usage. For instance, despite its lower expression in MCF-7 cells, TCF12 was significantly recruited to the central constituent of the SE, carrying an ERE and TCF motif (Figure 4C,D). This representative region clearly demonstrated how Ishikawa-specific factors localized together and how their TFBSs were separated from those of MCF-7-specific TFs (Figure 4D). Moreover, in Ishikawa cells, ERα mostly occupied binding sites together with its collaborating factors. By contrast, in MCF-7 cells, ERα binding events were much more isolated.

In order to measure the extent of ERα autonomy, we compared the number of SE constituents bound by at least two collaborating TFs that were present in MCF-7 or Ishikawa cells (Figure 4E). Within the 99 shared SEs, 47% of MCF-7-specific and 25% of Ishikawa-specific SE constituents were bound by a maximum of one TF other than ERα, which implies that in Ishikawa cells ERα typically requires several collaborating TFs for effective binding, while in MCF-7 cells this is optional. Another tendency observed at the *WWC1* locus was the opposed binding strength between ERα and its collaborating factors in Ishikawa cells (Figure 4C,D). In order to test this finding, we sorted protein densities by the ratio of ERα and TEAD4 signals (Supplementary Figure S5B). Indeed, the strongest ERα peaks were mostly established on EREs and typically lacked collaborating factors, while co-occurrence with strong cofactor binding resulted in moderate recruitment of ERα. This was in agreement with the results of correlation analyses, indicating the significance of DNA-protein and protein-protein interactions (Figure 3C, Supplementary Figure S4A). In the case of MCF-7-specific TFs, we observed a high ratio of direct ERE binding and a positive correlation between protein enrichments (Supplementary Figure S5C). Altogether, these results also supported our model (Figure 3D), showing that DNA binding by even one of the examined TFs was able to promote the recruitment of other TFs that might be part of a cell line-specific protein complex.

**Figure 5:**
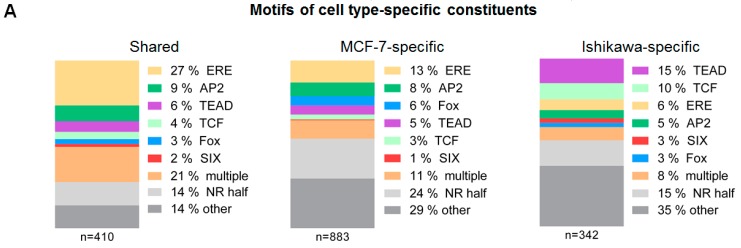

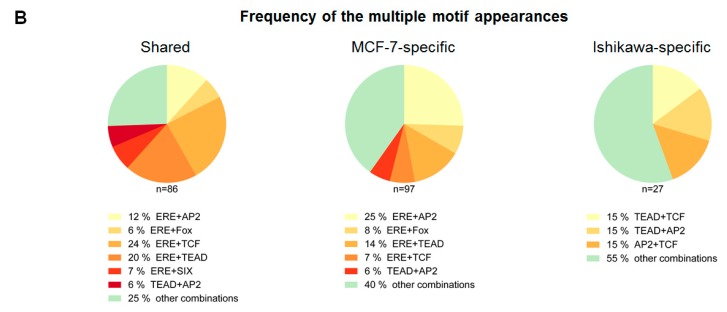
Motif composition within the cell line-specific constituents of the 99 shared SE regions. (**A**) The proportion of investigated DNA motifs within the 99 shared ERα-driven SEs visualized on three bar charts (stacked up to 100%) and classified according to Figure 4B. (**B**) Pie charts represent the frequency of the top multiple motif appearances defined on panel A.

In general, we found only one or two putative response element(s) per peak, and in some cases, none of the motif matrices used could be identified (Figure 4C). Within the common SE regions, the shared binding sites were dominated by ERE alone (27%) or in combination with other motifs (21%, concomitant presence of multiple motifs) and NR half-sites (14%) (Figure 5A,B). MCF-7-specific ERα binding sites showed a similar motif distribution but had a considerably higher proportion of NR half-sites (24%) and other motifs (29%). Alternatively, in Ishikawa cells, compared to ERE, TEAD (15%), TCF (10%), and NR half motifs (15%) dominated (Figure 5A,B). Notably, Ishikawa-specific SEs were typically bound by ERα at EREs not only in Ishikawa but also in MCF-7 cells, whereas MCF-7-specific SEs were poorly bound in Ishikawa cells (Supplementary Figure S6A,B). This result is consistent with the notion that while ERα is dominantly recruited to EREs or NR half-sites in MCF-7 cells, in Ishikawa cells ERα can also be recruited by TEAD4 and/or TCF12 (Figure 5A,B).

### 2.5. Common ERα-driven SEs Generate Similar Transcriptional Output in MCF-7 and Ishikawa Cells

**Figure 6:**
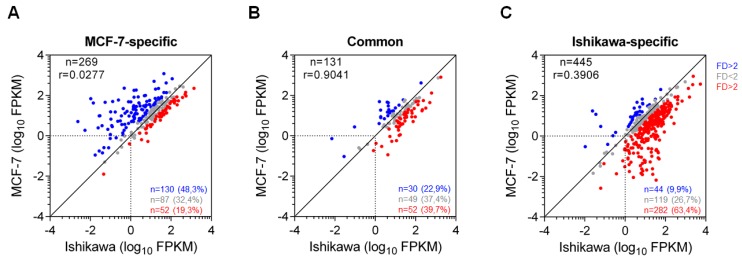
Genes regulated by shared SEs show identical expression in MCF-7 and Ishikawa cells. (**A**–**C**) Scatter plots showing the expression levels of genes closest to the MCF-7-specific (**A**), common (**B**) and Ishikawa-specific (**C**) ERα-driven SEs. Grey dots represent genes within a 2-fold difference (FD) range between both replicates of MCF-7 and Ishikawa cells; blue and red dots represent genes that exceed this range and are specific to MCF-7 or Ishikawa cells, respectively. The plots contain the number of the genes represented by blue, red or grey dots and the percentage values relative to the total gene number.

In the next step, we investigated the transcriptional outputs, namely, the maintained expression levels of the genes possibly regulated by cell line-specific and shared SEs identified in both cell lines upon E2 treatment (Ishikawa cells upon 240 min and MCF-7 cells upon 320 min of E2 treatment) (Figure 6A–C). 

Notably, the annotation of regulatory sites to proper genes represents a limitation to any study without using e.g., 3C-based methods, and we did not consider transient gene induction events. According to the main characteristic of SEs (these cause high expression), an asymmetric distribution pattern shifted to MCF-7-specific genes was observed for MCF-7-specific SEs. Nevertheless, several genes showed similar gene expression in both cell lines (<2-fold difference), and a number of genes showed higher expression in Ishikawa cells than in MCF-7 cells (>2-fold difference), although the enhancers’ coverage by ERα was not high enough to call them SEs in Ishikawa cells (Figure 6A). The high expression in Ishikawa cells around MCF-7-specific SEs might be due to the presence or absence of different factors (e.g., repressors) or even technical difficulties in SE prediction or gene annotation. A similar phenomenon with an opposite pattern in SE occupancy and gene expression output was also observed for Ishikawa-specific SE-related genes (Figure 6C), genes regulated by shared SEs showed similarly high expression in MCF-7 and Ishikawa cell lines, even though these had largely different sets of collaborative factors (Figure 6B).

## 3. Discussion

As SEs are responsible for cell identity, understanding their mechanism of action is indispensable for improving our knowledge of the regulation of gene expression in general and cellular identity in particular [24]. The recruitment and activation of a dominant TF represents a key step in the formation of SEs, and this recruitment is largely determined by the presence of specific DNA sequences. In addition to these DNA sequences, also known as motifs, which will later result in TF binding, the activating complexes are generated from gathered TFs; moreover, these complexes and protein-DNA interactions are stabilized by additional protein-protein interactions [15].

Previous studies focused mostly on changes in SEs during differentiation; here we examined how SE regions driven by the same master TF behave in two different cancer cell lines. We identified 392 ERα-driven SEs from the breast cancer-derived MCF-7 cells and 618 ERα-driven SEs from the endometrial cancer-derived Ishikawa cells. By examining their unique components, the first evidence was that despite a small overlapping subset, which likely involves conserved binding sites driven by strong EREs, cell type-specific sites indicated the presence of quite different TF binding motifs. These motifs were indeed bound by their recognition proteins, and through protein-protein interactions, some of them have a great affinity for each other. Moreover, certain TF partners might make ERα a hormone-sensitive coregulator, which increases TF binding affinity upon ligand treatment. However, additional investigations using, for example, mass spectrometry, protein interaction assays, and structural biochemical techniques are needed to identify all major components and oligomers of these cell line-specific complexes.

Although in the MCF-7 cell line ERα-dominated SEs are also modulated by the well-known FoxA1 and AP2γ TFs, as described previously, ERα has no coequal TF partner. In Ishikawa cells, we assumed the existence of at least a tripartite protein complex composed of TEAD4, TCF12, FoxM1, or C/EBPβ in which FoxM1 might have the highest affinity for ERα. However, the role of FoxM1 might be only secondary without its own response elements, and there can be additional TFs playing roles in the assembled regulatory complexes. Of course, the overall structure of a complex based on ChIP-seq data cannot be determined, and further specific experiments supporting this model should be applied. In this study, we carried out a theoretical investigation by using a large publicly available data set derived from two cell lines; however, the proteins identified based on their DNA motifs are indeed parts of a complex acting at SEs and some of them (TEAD4 or TCF12) might have a so-far unknown pioneer role.

Out of the predicted 392 (MCF-7) and 618 (Ishikawa) ERα-driven SEs, only 99 regions overlapped in the two cell lines where ERα was recruited in an alternating fashion, depending on the presence of cell line-specific co-factors and their motif(s). In Ishikawa cells, three-quarters of these ERα binding sites required the presence of at least two collaborative factors. By contrast, in MCF-7 cells, only half of the binding sites showed the presence of both FoxA1 and AP2γ or even TEAD4/TCF12. This observation may be explained by ERα binding directly to DNA through the ERE, and FoxA1 or AP2γ rather facilitates and/or stabilizes their presence in MCF-7 cells. By contrast, in Ishikawa cells, except for strong canonical EREs, ERα binding occurs almost exclusively through DNA binding collaborating factors. Overall, we observed that depending on the expressed collaborating TFs, ERα behaves in very different ways. One strong ERE might be sufficient for ERα binding but it is basically insufficient for regulation. Nearby motifs—even within the same enhancer or distant ones within the enhancer cluster—will determine the functional ERα TFBSs, and ultimately the whole cistrome. These results explain how gene regulation is carried out by a combination of DNA sequences selected by a combination of expressed TFs. This is a two-level combinatorial code of regulation further supported by specific co-regulators establishing permissive chromatin environment and chromatin interactions.

Overall, the results confirm the importance of the examined SE-bound genomic regions and the genes affected by them. MCF-7-specific ERα-driven SEs indeed regulated genes with pivotal roles in breast cancer (e.g., *BCAS3*, as an amplified and overexpressed gene and *ZNF217*, the candidate organizer of repressive histone modifiers) (highlighted in blue in Supplementary Figure S7A) and altered the expression of genes regulated by Ishikawa-specific SEs, which were typically specific for endometrial cancer (e.g., *ANXA2* and *ATF4*) (highlighted in red in Supplementary Figure S7B) [48,49,50,51]. However, some genes were not specifically associated with these two tumor types, but it may be assumed that these play important roles as they are regulated by SEs. Importantly, the genes regulated by common SEs showed similar expression patterns in both MCF-7 and Ishikawa cells even though these were supported by different collaborating factors and most of these commonly regulated genes are associated with poor prognosis or metastatic conditions (e.g., *AZIN1*, *KRT8*, *KRT19*, *SLC9A3R1*, *HES1*, and *CXXC5*), further demonstrating the importance of understanding the function of strong/canonical regulatory elements or complete units, such as SEs (highlighted in grey in Supplementary Figure S7C) [52,53,54]. Our results suggest that cell type-specific SEs regulate genes with high expression levels, and most of these are indeed linked to (breast and/or endometrial) cancerous processes. Furthermore, the complete or partial deletion of these units may influence the expression of oncogenes so these can be targets of future investigations.

Although several studies reported that different cell types had different transcription profiles and specific TF pools, the essence of our hypothesis is that in different cell types, shared SEs can generate very similar transcriptional events even though these shared SEs are built from various individual enhancers based on the cell type-specific TF profile. Our proposal is that these shared SEs have DNA-encoded responsiveness towards different subsets of TFs, and these behave like *bona fide* multi-tools of the genome.

Our study has several limitations. The results rely on the super-enhancer-regulated genomic regions, not the whole genome is studied; and the cancer cell lines used for the study are not tested to copy number alterations. In addition, instead of early and transient gene expression changes that are difficult to handle, we focused on maintained, steady-state gene expression levels upon a longer period of time in both cell lines. In order to verify the presented cell-type-specific protein-protein interactions further biochemical and genomic methods are required. One of the major limitations of the study is that our bioinformatic investigations were performed on breast- and endometrial cancer cell lines. For a better understanding of the tissue specificity of SEs, animal studies would be needed. 

Besides the limitations highlighted, we believe that our investigations provide a new level of understanding of the plasticity of transcription regulation with potential clinical implications. Based on the presented results, further investigations can be initiated that could widen our understanding of the effects and side-effects of hormonal treatments in these two endocrine dependent tissues, namely breast and endometrium, both of these being of major interest in women’s health.

## 4. Materials and Methods 

### 4.1. Data Selection

Raw ChIP-seq, DNase-seq, and RNA-seq data were downloaded from the Gene Expression Omnibus (GEO). As we used data from ECC-1 isolates of ATCC (American Type Culture Collection, Manassas, VA, USA) that were genotyped as Ishikawa cells [55]; we therefore referred to them as Ishikawa cells. Detailed information about the selected data (including GEO identifiers and references for the publications where these were first published) is included in Supplementary Table S1A–C.

### 4.2. ChIP-seq Analysis

Raw sequence data were analyzed uniformly with an updated version of our previously published computational pipeline [56] as follows: raw sequence reads were aligned to the hg19 reference genome assembly (GRCh37) by using the Burrows-Wheeler Alignment (BWA) tool (v07.10) [57], then BAM files were generated with SAMtools (v0.1.19) [58]. Coverage files were created by the *makeUCSCfile.pl* script of the Hypergeometric Optimization of Motif EnRichment (HOMER) package (v4.2) [59], and peaks were predicted using the Model-based Analysis of ChIP-Seq (MACS2) tool (v2.0.10) with *-callpeak* parameter [60]. In order to remove the artifacts from the predicted peaks, we used the blacklisted genomic regions of the Encyclopedia of DNA Elements (ENCODE) [61].

Reads Per Kilobase per Million mapped reads (RPKM) values were calculated on the ±50-bp regions relative to the peak summits by using the *coverageBed* program of BedTools (v2.23.0) [62]. The number of overlapping peaks and regions was defined by using the DiffBind package (v1.2.4) in R [63]. Read distribution (RD) heat maps were generated by *annotatePeaks.pl* with *-hist 50* and *-ghist* parameters (HOMER). Coverage values for average protein density heat maps were calculated on the summit positions of the RD plots.

### 4.3. Super-Enhancer Prediction

Super-enhancers were predicted from the E2-treated ERα ChIP-seq samples applying the HOMER’s *findPeaks.pl* script and the *-style super* parameter. In order to generate a “super-enhancer plot”, we used the *-superSlope -1000* parameter and the thus generated “Normalized Tag Count” values were plotted. Tag counts (rpm/bp; reads per million per base pair) of the ERα (super-)enhancers were ranked by their ChIP-seq coverage. The definition of super-enhancers was based on the original strategy, where the outstandingly “active” enhancers or broader regions in which enhancers are closer than 12.5 kb to each other are over slope 1 in the rank order.

### 4.4. Motif Analysis

Motif enrichment analysis was carried out by the *findMotifsGenome.pl* script of HOMER. It was performed on the ±100 bp flanking regions of the peak summits. The lengths for the motif search were 10, 12, 14, and 16 bp. *p*-values were calculated by comparing the enrichment within the target regions and that of a random set of regions (background) generated by HOMER.

For the motif distribution plot, motif matrices (shown in Supplementary Figure S2A) were mapped in 30-bp windows within 1.5-kb frames relative to the ERα peak summits using *annotatePeaks.pl* with *-mbed* parameter, BEDtools, and other command-line programs. The clustering of motif distribution patterns was done by Cluster 3.0. The top motif score for each examined region was determined by *annotatePeaks.pl* with *-mscore* parameter. Thresholds of the plotted scores were selected before the last markedly high peak of the motif score distribution.

### 4.5. DNase-seq Analysis

The primary analysis of DNase-seq data was carried out as described for ChIP-seq data analysis.

### 4.6. RNA-seq Analysis

Raw sequence reads were aligned to the hg19 reference genome assembly (GRCh37) by using TopHat (v2.0.7). The Fragments Per Kilobase of transcript per Million mapped reads (FPKM) values were calculated by Cufflinks (v2.0.2) with default parameters [64].

### 4.7. Gene Annotation

Super-enhancers were extended to ±100-kb relative to their center. The highest expressed protein-coding gene (at 240 and 320 min of E2 treatment of Ishikawa and MCF-7 cells, respectively) the promoter of which overlapped with an extended region was assigned to the related super-enhancer. If an extended region could not be annotated to any gene, the closest protein-coding gene with at least 1 FPKM expression value was assigned to it by using the PeakAnnotator program [65].

### 4.8. Visualization

Read distribution, average protein density, and correlation heat maps were plotted by Java TreeView (v1.1.6r4) [66]. Area-proportional Venn diagrams were produced by BioVenn [67]. Box plots, scatter plots, bar charts, and histograms were created with GraphPad Prism 6. Coverage files were visualized by Integrative Genomics Viewer v2.4.16 (IGV) [68].

## Supplementary Materials

Supplementary materials can be found at https://www.mdpi.com/1422-0067/21/5/1630/s1.

## Abbreviations

ERα	estrogen receptor alpha
ERE	estrogen response element
E2	estradiol (17β-estradiol)
H3K27ac	acetylation at the 27th lysine residue of the histone H3 protein
NR	nuclear receptor
SE	super-enhancer
TF	transcription factor
TFBS	transcription factor binding site
