# Supplementary material for: Alternatively Constructed Estrogen Receptor Alpha-Driven Super-Enhancers Result in Similar Gene Expression in Breast and Endometrial Cell Lines
Source: Int J Mol Sci. 2020 Feb 27;21(5):1630. doi: 10.3390/ijms21051630 (PMC7084573; doi:10.3390/ijms21051630)
Supplement: Supplementary file 1 [file ijms-21-01630-s001.pdf]

Supplemental information

Alternatively constructed estrogen receptor alpha-driven super-enhancers result in similar gene expression in breast and endometrial cell lines

Dóra Bojcsuk, Gergely Nagy and Bálint László Bálint

SUPPLEMENTARY FIGURES (page 2-10)

Supplementary Figure 1. Enrichment of active chromatin marks and regulatory factors follows ERα binding patterns in MCF-7 and Ishikawa cells.

Supplementary Figure 2. Transcription factor binding correlates well with response element strength.

Supplementary Figure 3. The gene expression levels of putative TF families.

Supplementary Figure 4. Correlation between transcription factor bindings and presence of their response elements.

Supplementary Figure 5. Shared ERα-driven super-enhancers are driven by different motifs in MCF-7 and Ishikawa cells.

Supplementary Figure 6. Cell type-specific ERα-driven super-enhancers are driven by different motifs in MCF-7 and Ishikawa cells.

Supplementary Figure 7. The identified SEs indeed regulate genes with pivotal role in cancer and cell fate.

SUPPLEMENTARY TABLE (page 11)

Supplementary Table 1. Table of used next generation sequencing data to characterize super-enhancers.

SUPPLEMENTAL REFERENCES (page 12)

Supplementary Figure 1.

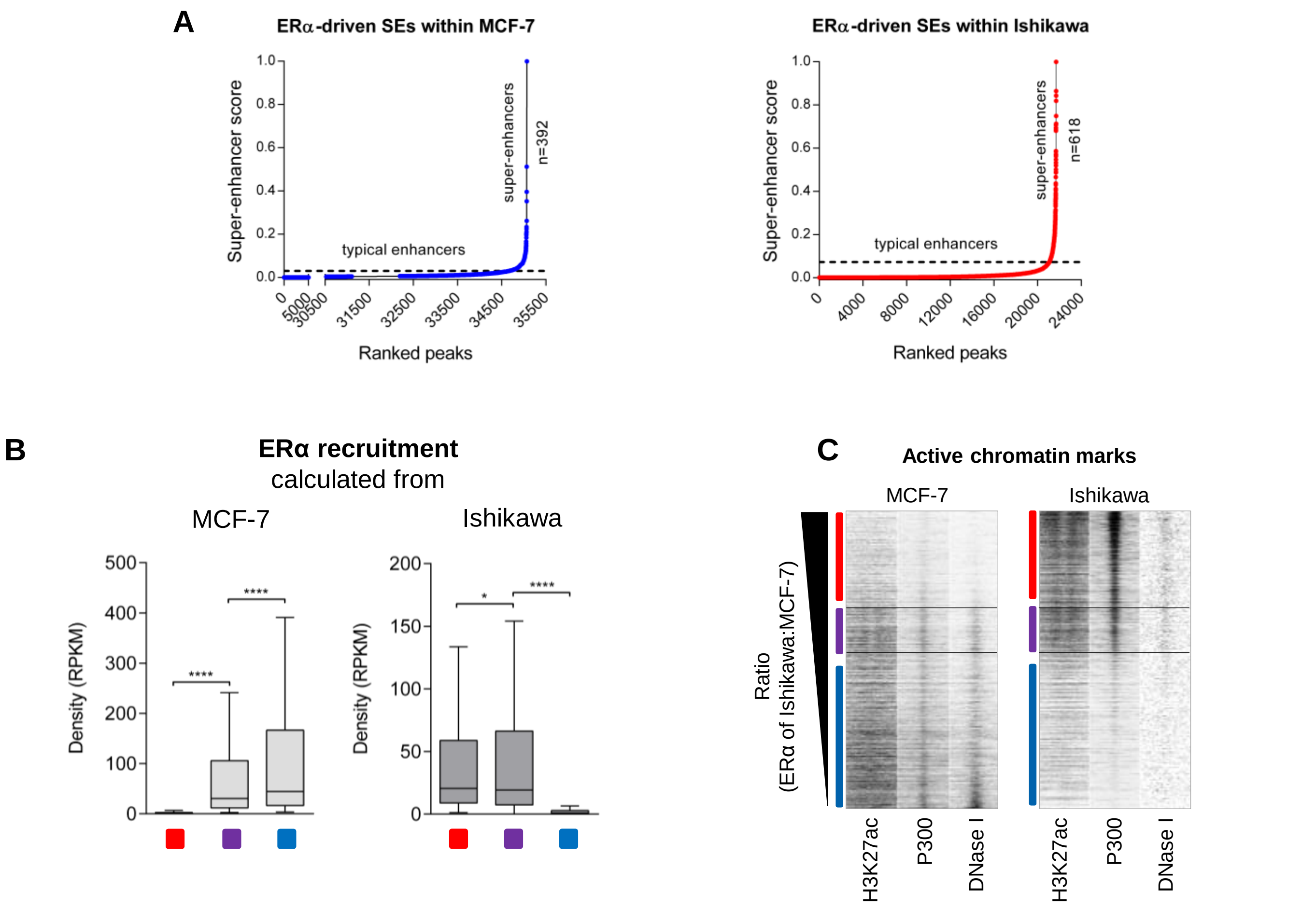

D

Iskihawa-specific SE constituents

Total target seq.: 2,137

P-value	Target %	Bg %	Motif
1e-110	23.21 %	7.58 %	NR half
1e-71	10.44 %	2.44 %	TCF
1e-68	12.73 %	3.65 %	TEAD
1e-44	9.83 %	3.17 %	AP-1
1e-40	8.94 %	2.91 %	SIX

Shared SE constituents

Total target seq.: 858

P-value	Target %	Bg %	Motif
1e-256	34.38 %	2.05 %	ERE
1e-47	27.97 %	10.14 %	DR 0
1e-36	13.40 %	3.21 %	DR(-)1
1e-33	11.07 %	2.40 %	DR 1

MCF-7-specific SE constituents

Total target seq.: 3,861

P-value	Target %	Bg %	Motif
1e-641	30.04 %	4.05 %	ERE
1e-510	59.08 %	22.77 %	NR half
1e-74	7.95 %	2.31 %	Fox
1e-45	8.52 %	3.54 %	AP2

Supplementary Figure 1. Enrichment of active chromatin marks and regulatory factors follows ERα binding patterns in MCF-7 and Ishikawa cells.

(Legends are available on the next page.)

Supplementary Figure 1. Enrichment of active chromatin marks and regulatory factors follows ERα binding patterns in MCF-7 and Ishikawa cells.

(A) The definition of ERα-driven SEs in MCF-7 and Ishikawa cell lines. Groups of enhancers (or even single enhancers) over slope 1 were considered to be SEs. **(B)** Box plots showing ERα recruitment within Ishikawa-specific, shared and MCF-7-specific clusters. RPKM (reads per kilobase per million mapped reads) values were calculated on the summit ± 50-bp regions of the ERα peaks, separately from the MCF-7 and Ishikawa ChIP-seq samples. The boxes represent the first and third quartiles, the horizontal lines indicate the median RPKM values and the whiskers indicate the 10th to 90th percentile ranges. Paired t-test, * significant at $P < 0.05$, ** at $P < 0.01$, *** at $P < 0.001$, **** at $P < 0.0001$. **(C)** Read distribution plots of H3K27ac and P300 ChIP-seq and DNase-seq (DNase I) data in MCF-7 and Ishikawa cell lines upon vehicle treatment relative to the ERα SE constituents in 2-kb frames in the same order as introduced in Figure 2A. **(D)** Detailed motif enrichment results within the ERα peaks of the three clusters (related to Figure 2B). *P*-values and target and background (Bg) percentages are included for each motif.

Supplementary Figure 2.

A

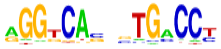

IR3 (ERE)
>RGGTCACNGTGACCTK
score: 8.046524

0.491	0.042	0.372	0.095
0.098	0.001	0.796	0.105
0.046	0.032	0.894	0.028
0.113	0.112	0.260	0.515
0.011	0.852	0.098	0.039
0.811	0.032	0.077	0.080
0.133	0.432	0.281	0.154
0.231	0.256	0.288	0.224
0.157	0.288	0.464	0.091
0.090	0.102	0.035	0.773
0.042	0.101	0.856	0.001
0.547	0.263	0.091	0.099
0.001	0.929	0.032	0.038
0.126	0.768	0.001	0.105
0.098	0.295	0.067	0.540
0.116	0.214	0.368	0.302

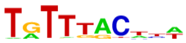

Fox(A1)
>WAAGTAAACA
score: 7.412125

0.446	0.053	0.014	0.488
0.487	0.100	0.323	0.090
0.517	0.027	0.139	0.317
0.084	0.032	0.846	0.038
0.010	0.239	0.013	0.738
0.714	0.213	0.018	0.055
0.864	0.055	0.027	0.054
0.953	0.011	0.025	0.011
0.025	0.630	0.012	0.333
0.928	0.007	0.013	0.052

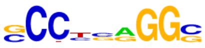

AP2(γ)
>SCCTSAGGCHATD
score: 8.347576

0.001	0.491	0.507	0.001
0.001	0.997	0.001	0.001
0.001	0.937	0.001	0.061
0.029	0.342	0.082	0.547
0.095	0.456	0.423	0.026
0.598	0.020	0.359	0.023
0.025	0.001	0.973	0.001
0.001	0.001	0.997	0.001
0.001	0.608	0.388	0.003
0.329	0.323	0.154	0.194
0.440	0.128	0.168	0.263
0.173	0.210	0.177	0.441
0.205	0.168	0.325	0.302

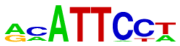

TEAD(4)
>NNACATTCCT
score: 7.558580

0.256	0.310	0.162	0.272
0.156	0.266	0.273	0.305
0.595	0.001	0.403	0.001
0.300	0.640	0.059	0.001
0.997	0.001	0.001	0.001
0.001	0.001	0.001	0.997
0.001	0.001	0.001	0.997
0.053	0.945	0.001	0.001
0.001	0.814	0.001	0.184
0.368	0.001	0.001	0.630

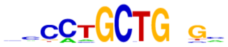

TCF(12)
>CCCCTGCTGKGM
score: 8.784753

0.167	0.457	0.203	0.174
0.174	0.559	0.100	0.167
0.022	0.797	0.086	0.095
0.219	0.779	0.001	0.001
0.132	0.108	0.001	0.759
0.001	0.011	0.987	0.001
0.001	0.997	0.001	0.001
0.014	0.043	0.001	0.942
0.001	0.001	0.977	0.021
0.223	0.146	0.246	0.385
0.001	0.113	0.766	0.120
0.386	0.428	0.076	0.109

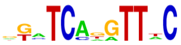

SIX(1)
>GKVTCADRTTWC
score: 8.095056

0.181	0.094	0.565	0.160
0.091	0.039	0.481	0.388
0.400	0.292	0.265	0.042
0.001	0.001	0.001	0.997
0.001	0.997	0.001	0.001
0.830	0.001	0.081	0.088
0.278	0.079	0.337	0.306
0.380	0.069	0.484	0.067
0.001	0.001	0.001	0.997
0.001	0.001	0.147	0.851
0.517	0.049	0.001	0.433
0.001	0.997	0.001	0.001

B

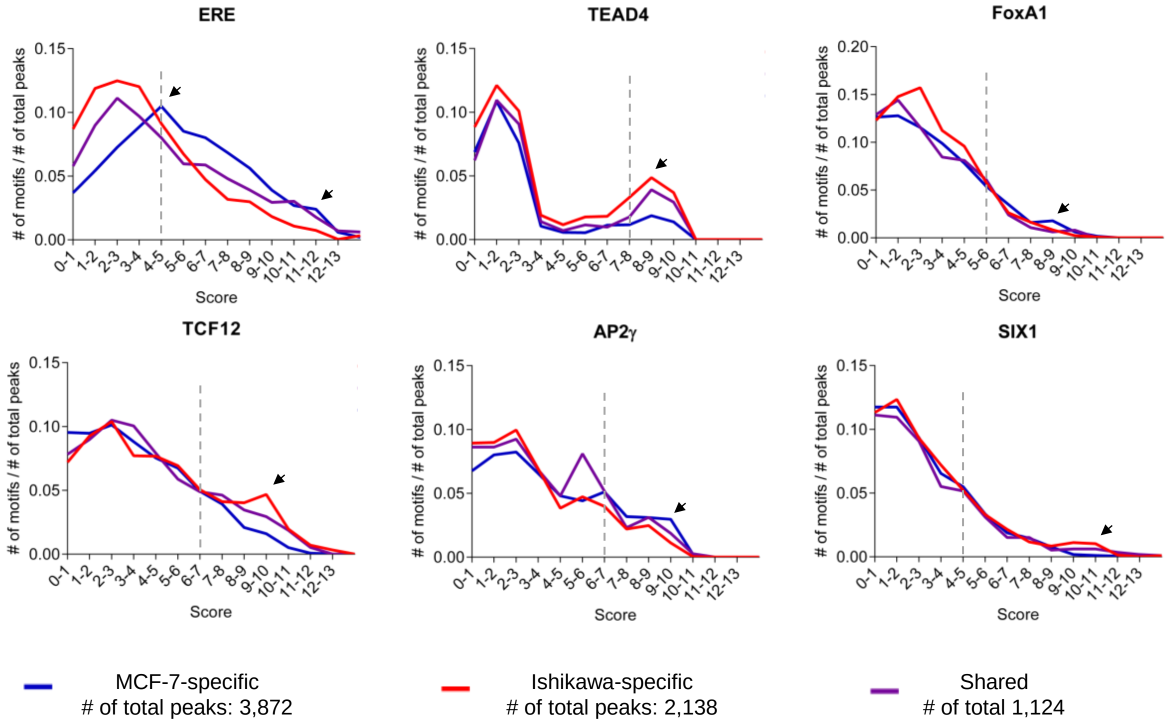

MCF-7-specific
# of total peaks: 3,872

Ishikawa-specific
# of total peaks: 2,138

Shared
# of total 1,124

Supplementary Figure 2. Transcription factor binding correlates well with response element strength.

(Legends are available on the next page.)

Supplementary Figure 2. Transcription factor binding correlates well with response element strength.

(A) The logos and matrices of enriched ERE, Fox, AP2, TCF, TEAD and SIX motifs used for mapping. **(B)** Histograms showing the frequency (#) of motifs depending on their score. The total number of motifs was divided with the given cluster size. Red, blue and purple lines represent Ishikawa-specific, MCF-7-specific and common ERα peaks, respectively. Dashed lines indicate the score threshold used for the motif strength analysis shown in Figure 2D, and arrows show motif enrichments specific to a cluster.

Supplementary Figure 3.

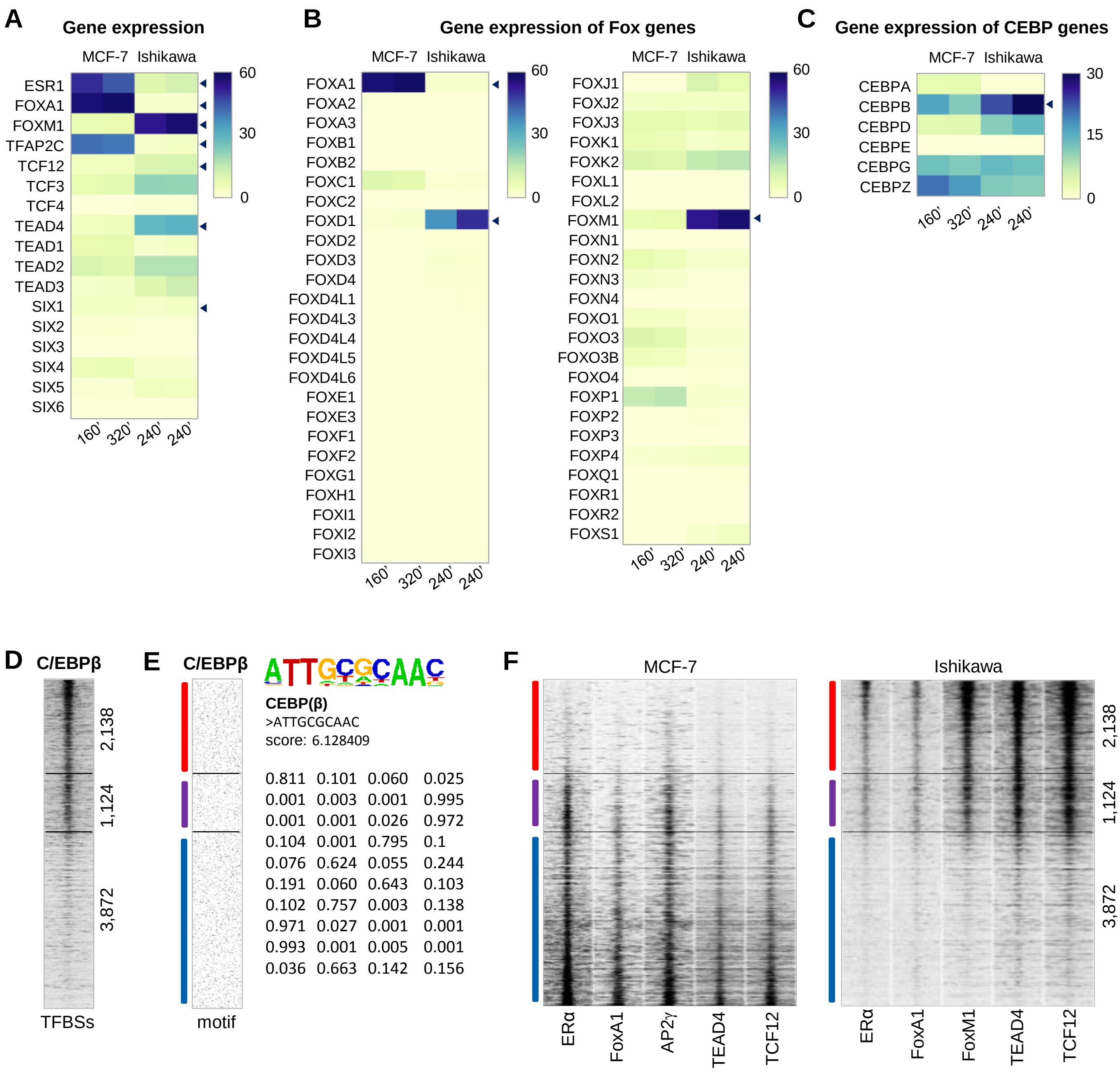

Supplementary Figure 3. The gene expression levels of putative TF families.

(A, B, C) The gene expression levels of putative regulator TF families **(A)** and the whole Fox **(B)** and CEBP **(C)** families in MCF-7 and Ishikawa cells. MCF-7 cells were treated with 10 nM E2 for 160 or 320 min, and Ishikawa cells were treated with 10 nM E2 for 240 min. Fragments per kilobase per million mapped reads (FPKM) values are shown. **(D)** Read distribution plot of CEBPβ coverage (upon vehicle treatment) was calculated from Ishikawa cells. **(E)** The heat map showing the CEBP motifs in the 1.5-kb frame. **(F)** Read distribution plots of the indicated TFs in MCF-7 and Ishikawa cells upon vehicle treatment in 2-kb frames. In the cases of panel D, E and F, coverage values and motifs were calculated around the summit position of ERα-driven SE constituents in the same order as introduced in Figure 2A.

Supplementary Figure 4.

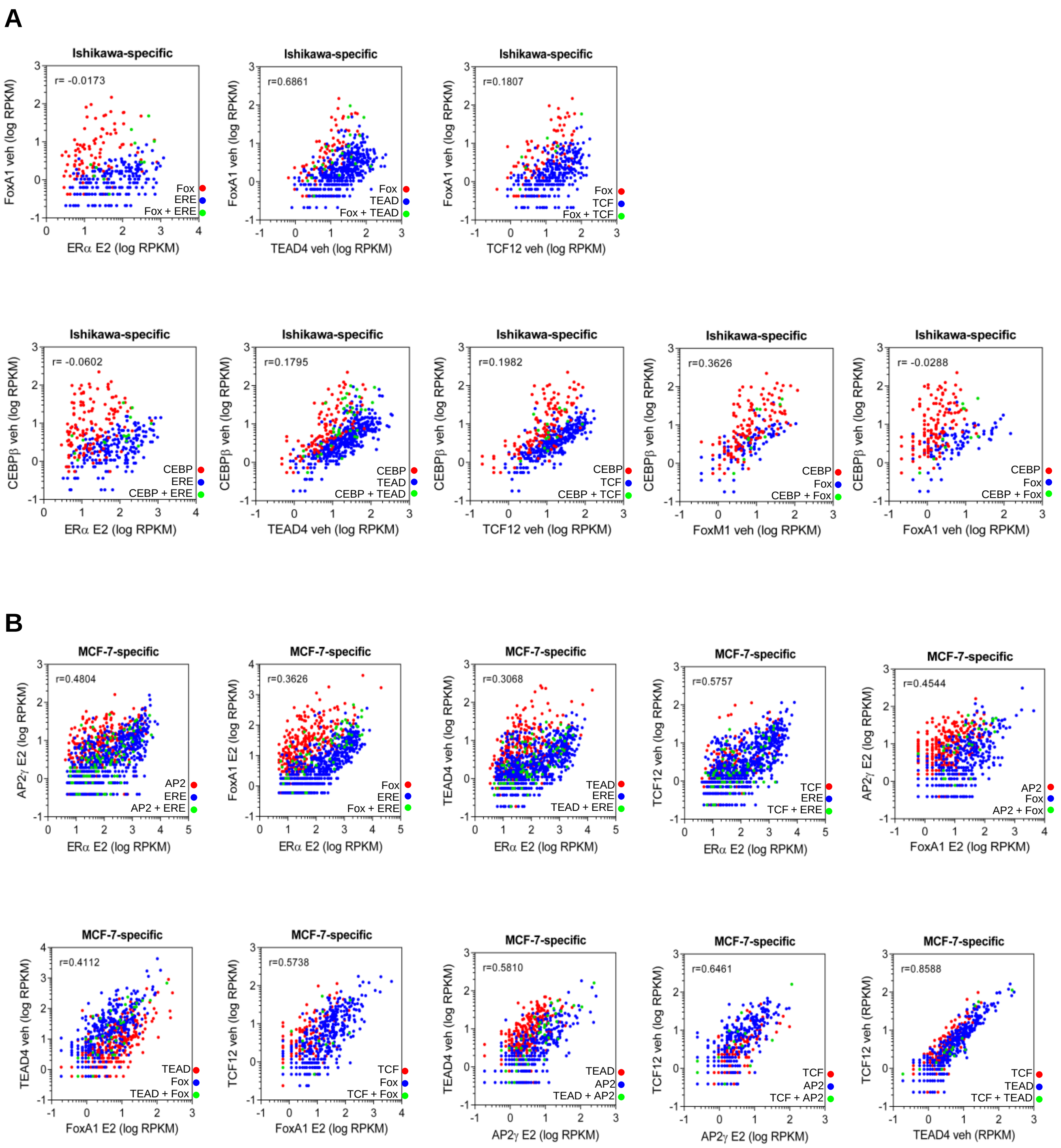

Supplementary Figure 4. Correlation between transcription factor bindings and presence of their response elements.

(A, B) Scatter plots showing the densities of the indicated TFs (upon vehicle [veh] or E2 treatment) on their DNA-binding motifs within the MCF-7- **(A)** and Ishikawa-specific **(B)** ERα-driven SE constituents. Red and blue dots represent protein binding at the specific single motif, and green dots represent protein binding at a region with the motifs of both examined TFs.

Supplementary Figure 5.

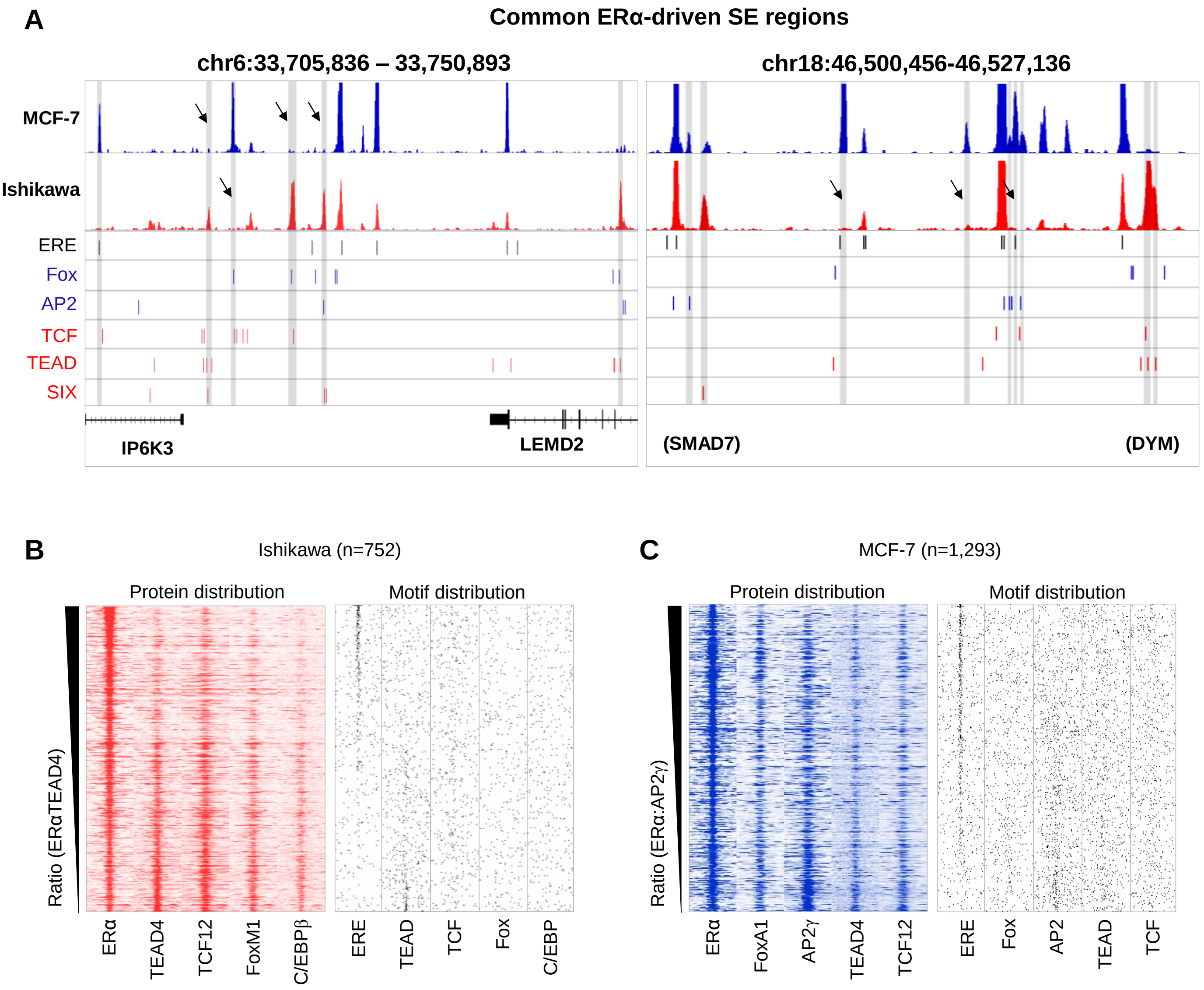

Supplementary Figure 5. Shared ERα-driven super-enhancers are driven by different motifs in MCF-7 and Ishikawa cells.

(A) Integrative Genomics Viewer snapshots of ERα ChIP-seq coverage on overlapping (shared) ERα-driven SEs in MCF-7 and Ishikawa cells upon E2 treatment. The interval scale is 50. The matrix of ERE, Fox, AP2, TCF, TEAD and SIX motifs was mapped within the summit ± 50-bp regions of the ERα peaks, and the indicated putative elements are represented as thin lines (bottom). Peaks marked with arrows and highlighted in grey show different binding patterns between MCF-7 and Ishikawa cells. **(B, C)** Read distribution plots showing the TF densities calculated on the 2-kb frame of the 752 Ishikawa and 1,293 MCF-7-specific SE constituents depicted on Figure 4E. Peaks were sorted based on the ratio of RPKM values calculated from ERα and TEAD4 in Ishikawa cells **(B)** and from ERα and AP2γ in MCF-7 cells **(C)**. Motif distribution heat maps represented in the same order as determined by protein density ratios **(B, C)**.

Supplementary Figure 6.

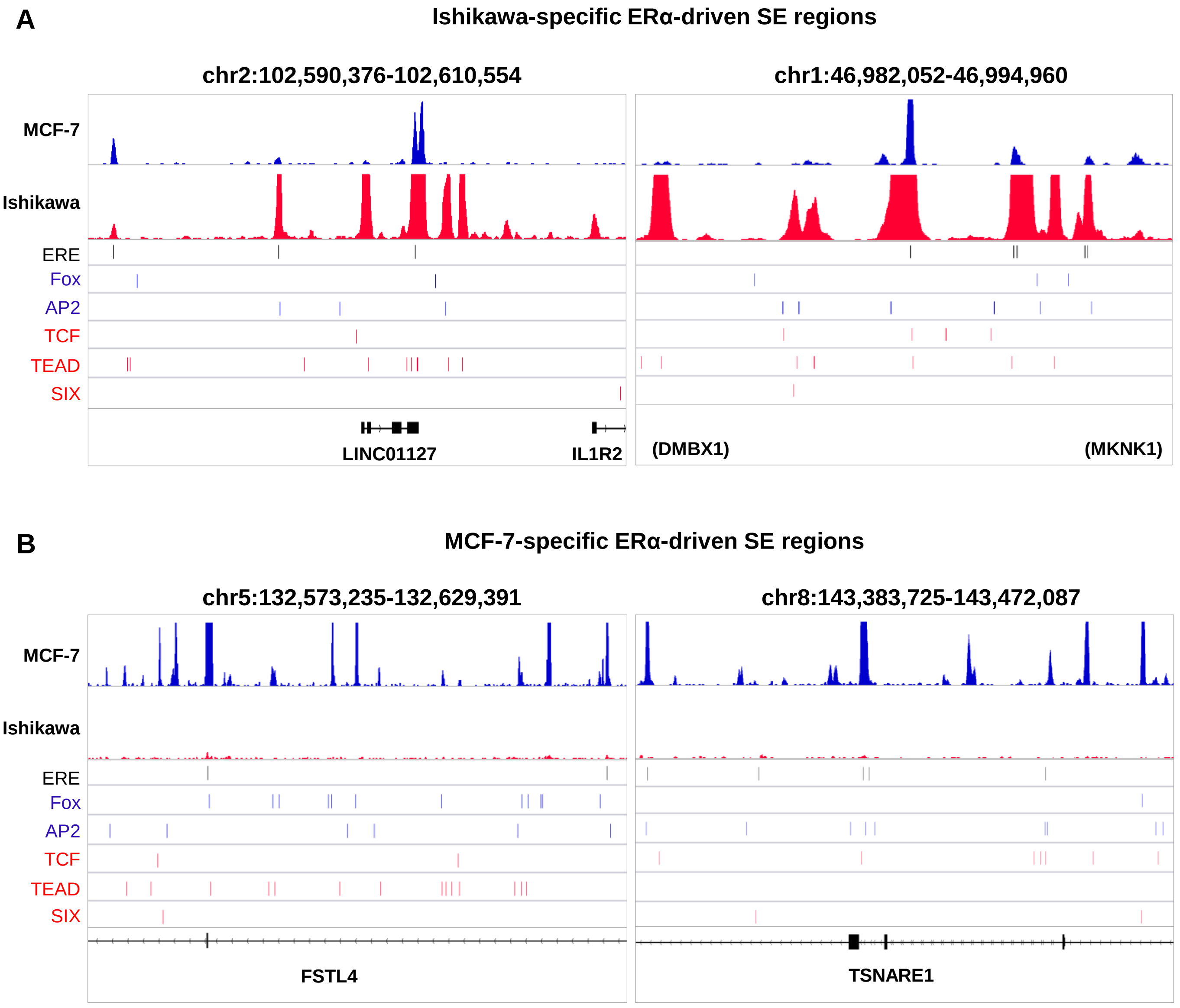

Supplementary Figure 6. Cell type-specific ERα-driven super-enhancers are driven by different motifs in MCF-7 and Ishikawa cells.

(A, B) Integrative Genomics Viewer snapshots of ERα ChIP-seq coverage on Ishikawa-specific **(A)** and MCF-7-specific **(B)** ERα-driven SEs in MCF-7 and Ishikawa cells upon E2 treatment. The interval scale is 50. The matrix of ERE, Fox, AP2, TCF, TEAD and SIX motifs was mapped within the summit ± 50-bp regions of the ERα peaks, and the indicated putative elements are represented as thin lines (bottom). Peaks marked with arrows and highlighted in grey show different binding patterns between MCF-7 and Ishikawa cells.

Supplementary Figure 7.

A

Top 10 highly expressed genes related to the MCF-7-specific SEs

MCF-7	Common	Ishikawa
XBP1	HSPB1	S100A10
ZNF217	CCND1	SPINT2
PARD6B	CTSD	C21orf33
NCOA3	SDC4	UBE2I
NMD3	CLDN4	METRNL
BCAS3	PDCD6	CAMTA1
EMP2	UBE2V1	LAMC1
PREX1	SPIN1	PODXL
SULF2	TOMM20	ECE1
LY6E	GINS2	C9orf3

B

Top 10 highly expressed genes related to the Ishikawa-specific SEs

MCF-7	Common	Ishikawa
NUCKS1	UBC	ANXA2
SLC38A2	H3F3B	ATF4
SIAH2	H3F3C	BSG
PPP4R2	CCND1	NDUFA11
CA12	TACSTD2	EIF5A
PPP1CB	RHOC	PPIF
MAP4K3	CXXC5	ASS1
ZNF281	TFRC	BANF1
DYRK2	ACTN1	EIF3G
ACAA2	TOP2A	CRIP2

C

Top 10 highly expressed genes related to the commonly regulated SEs

MCF-7	Common	Ishikawa
CPN2	KRT8	RNU86
LRRC15	KRT19	TUBB
EGR3	LSM3	GPX4
KCNK15	SLC9A3R1	MNF1
SYNJ2	TPD52L1	LAPTM4B
FOXK1	APRT	COX5A
PMEPA1	CXXC5	MDK
FMN1	TIMM23	SLC7A5
P2RY2	MRPS23	TRAPPC2L
RXRA	HES1	NME4

Supplementary Figure 7. The identified SEs indeed regulate genes with pivotal role in cancer and cell fate.

(A, B, C) Tables contain the top 10 highly expressed protein-coding genes regulated potentially by the cell type-specific **(B, C)** and the common **(D)** SEs. Genes were further divided (highlighted with blue, grey or red) depending on which cell type expressed it to a greater extent (as defined on Figure 6 A, B and C).

Supplementary Table 1.

A

GEO ID	Cell line	Predicted SEs	Peaks within SEs	Reference
GSM614610	MCF-7	392	4,042	(1)
GSM803422	Ishikawa	618	3,517	(2)

B

Experiment	Factor	Cell line	GEO ID (vehicle)	GEO ID (treated)	Reference
ChIP-seq	ERα	MCF-7	GSM614611	GSM614610	(1)
ChIP-seq	ERα	Ishikawa	GSM803421	GSM803422	(2)
ChIP-seq	FoxA1	MCF-7	GSM588929	GSM588930	(3)
ChIP-seq	FoxA1	Ishikawa	GSM803444	-	(2)
ChIP-seq	TCF12	MCF-7	GSM1010861	-	
ChIP-seq	TCF12	Ishikawa	GSM1010842	-	
ChIP-seq	TEAD4	MCF-7	GSM1010860	-	
ChIP-seq	TEAD4	Ishikawa	GSM1010885	-	
ChIP-seq	AP2γ	MCF-7	GSM1469997	GSM1469998	(4)
ChIP-seq	FoxM1	Ishikawa	GSM1010856	-	(2)
ChIP-seq	CEBPβ	Ishikawa	GSM1010802	-	(2)
ChIP-seq	H3K27ac	MCF-7	GSM1382472	-	(5)
ChIP-seq	H3K27ac	Ishikawa	GSM1635579	-	(6)
ChIP-seq	P300	MCF-7	GSM1470013	-	(4)
ChIP-seq	P300	Ishikawa	GSM1010759	-	(2)
DNase-seq	DNase I	MCF-7	GSM822390	-	(7)
DNase-seq	DNase I	Ishikawa	GSM1008597	-	(8, 9)
RNA-seq	-	MCF-7	-	GSM1533420	(10)
RNA-seq	-	MCF-7	-	GSM1533421	
RNA-seq	-	Ishikawa	-	GSM2453337	(8)
RNA-seq	-	Ishikawa	-	GSM2453338	

Supplementary Table 1. Table of used next generation sequencing data to characterize super-enhancers.

(**A**) Information about the ERα ChIP-seq samples used for the basic analysis. **(B)** Information about ChIP-seq, DNase-seq and RNA-seq samples used for the characterization of ERα-driven SEs.

Supplemental references

1. Schmidt,D., Schwalie,P.C., Ross-Innes,C.S., Hurtado,A., Brown,G.D., Carroll,J.S., Flicek,P. and Odom,D.T. (2010) A CTCF-independent role for cohesin in tissue-specific transcription. *Genome Res.*, **20**, 578–588. doi:10.1101/gr.100479.109

2. Gertz,J., Savic,D., Varley,K.E., Partridge,E.C., Safi,A., Jain,P., Cooper,G.M., Reddy,T.E., Crawford,G.E. and Myers,R.M. (2013) Distinct Properties of Cell-Type-Specific and Shared Transcription Factor Binding Sites. *Mol. Cell*, **52**, 25–36. doi:10.1016/j.molcel.2013.08.037

3. Tan,S.K., Lin,Z.H., Chang,C.W., Varang,V., Chng,K.R., Pan,Y.F., Yong,E.L., Sung,W.K., Sung,W.K. and Cheung,E. (2011) AP-2γ regulates oestrogen receptor-mediated long-range chromatin interaction and gene transcription. *EMBO J.*, **30**, 2569–81. doi:10.1038/emboj.2011.151

4. Liu,Z., Merkurjev,D., Yang,F., Li,W., Oh,S., Friedman,M.J., Song,X., Zhang,F., Ma,Q., Ohgi,K.A., *et al.* (2014) Enhancer Activation Requires trans-Recruitment of a Mega Transcription Factor Complex. *Cell*, **159**, 358–373. doi:10.1016/j.cell.2014.08.027

5. Brunelle,M., Nordell Markovits,A., Rodrigue,S., Lupien,M., Jacques,P.-É. and Gévry,N. (2015) The histone variant H2A.Z is an important regulator of enhancer activity. *Nucleic Acids Res.*, **43**, 9742–56. doi:10.1093/nar/gkv825

6. Zhang,X., Choi,P.S., Francis,J.M., Imielinski,M., Watanabe,H., Cherniack,A.D. and Meyerson,M. (2016) Identification of focally amplified lineage-specific super-enhancers in human epithelial cancers. *Nat. Genet.*, **48**, 176–182. doi:10.1038/ng.3470

7. He,H.H., Meyer,C.A., Chen,M.W., Jordan,V.C., Brown,M. and Liu,X.S. (2012) Differential DNase I hypersensitivity reveals factor-dependent chromatin dynamics. *Genome Res.*, **22**, 1015–25. doi:10.1101/gr.133280.111

8. Dunham,I., Kundaje,A., Aldred,S.F., Collins,P.J., Davis,C.A., Doyle,F., Epstein,C.B., Frietze,S., Harrow,J., Kaul,R., *et al.* (2012) An integrated encyclopedia of DNA elements in the human genome. *Nature*, **489**, 57–74. doi:10.1038/nature11247

9. Natarajan,A., Yardimci,G.G., Sheffield,N.C., Crawford,G.E. and Ohler,U. (2012) Predicting cell-type-specific gene expression from regions of open chromatin. *Genome Res.*, **22**, 1711–1722. doi:10.1101/gr.135129.111

10. Honkela,A., Peltonen,J., Topa,H., Charapitsa,I., Matarese,F., Grote,K., Stunnenberg,H.G., Reid,G., Lawrence,N.D. and Rattray,M. (2015) Genome-wide modeling of transcription kinetics reveals patterns of RNA production delays. *Proc. Natl. Acad. Sci.*, **112**, 13115–13120. doi:10.1073/pnas.1420404112
